# Late cardiac tamponade after a helix‐based active fixation leadless pacemaker implantation

**DOI:** 10.1002/joa3.12987

**Published:** 2024-01-02

**Authors:** Ryuki Chatani, Hiroshi Tasaka, Kenta Yoshida, Mitsuru Yoshino, Kazushige Kadota

**Affiliations:** ^1^ Department of Cardiovascular Medicine Kurashiki Central Hospital Kurashiki Japan

**Keywords:** cardiac tamponade, helix‐based active fixation, leadless pacemakerAveir‐VR, leadless pacemaker

## Abstract

Although the late cardiac tamponade in leadless pacemaker implantation (LPI) is rare, we encountered such an incident in patient with AVEIR‐VR™ system on hemodialysis and warfarinization. When LPI with active fixation system, we should aim for successful single‐attempt deployment using electrical premapping to prevent cardiac tamponade including the late phase.

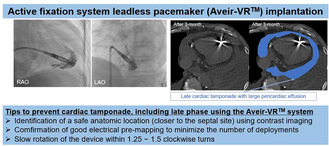

In leadless pacemaker implantation (LPI), acute cardiac tamponade must be avoided. An active fixation LPI system (Aveir‐VR™) is expected to reduce the cardiac tamponade by decreasing the number of attempts through contact mapping of electric parameters before fixation. Reports of late cardiac tamponade in LPI are very rare. Herein, we present the case, of a patient with sick sinus syndrome (SSS), who underwent LPI and was uneventfully discharged. However, 3 months after LPI, he was readmitted with late cardiac tamponade.

The patient was a 72‐year‐old man with a history of unprovoked pulmonary embolism (PE) and end‐stage renal disease (ESRD) on hemodialysis. He was hospitalized for presyncope to SSS type I. He was prescribed warfarin because of a history of PE. His body weight and body mass index were 70.4 kg and 24.5 kg/m^2^, respectively. Because he had both brachial artery and vein shunts for hemodialysis, LPI with Aveir‐VR™ (Abbott) was planned.

At the first attempt, Aveir‐VR™ was deployed on the lower mid‐septum; however, the threshold was unacceptable. At the second attempt, Aveir‐VR™ was successfully placed on the apical site (Figure 1). The procedure time was 96 min, and the patient was uneventfully discharged 3 days after LPI; the threshold was 0.25 V at 0.4 ms, R wave 10.5 mV, and impedance 1010 Ω. One month after LPI, his Aveir‐VR™ electrical parameters were not abnormal, and his ventricular pacing rate was 71% (VVI 50 mode). His pericardial effusion was not changed (Figure 2A,B). However, 106 days after LPI, he was admitted because of difficulty in hemodialysis owing to late cardiac tamponade (Figure 2C). No changes in Aveir‐VR™ electrical parameters were noted: the threshold was 0.75 V at 0.2 ms, R wave 5.5 mV, and impedance 600 Ω. His PT‐INR was 3.22, so warfarin was stopped, and the PT‐INR was reversed with a warfarin antagonist. Pericardial drainage was performed by puncture, and 600 mL of bloody pericardial effusion was drained. The drain was removed 2 days later, and after removal, pericardial effusion did not recur, no right ventricular perforation of an Aveir‐VR™ (Figure 2D) occurred, and he was discharged 4 days after drain removal. Warfarin remained discontinued after discharge, and there was no recurrence of pericardial effusion 1 month after discharge.

**FIGURE 1:**
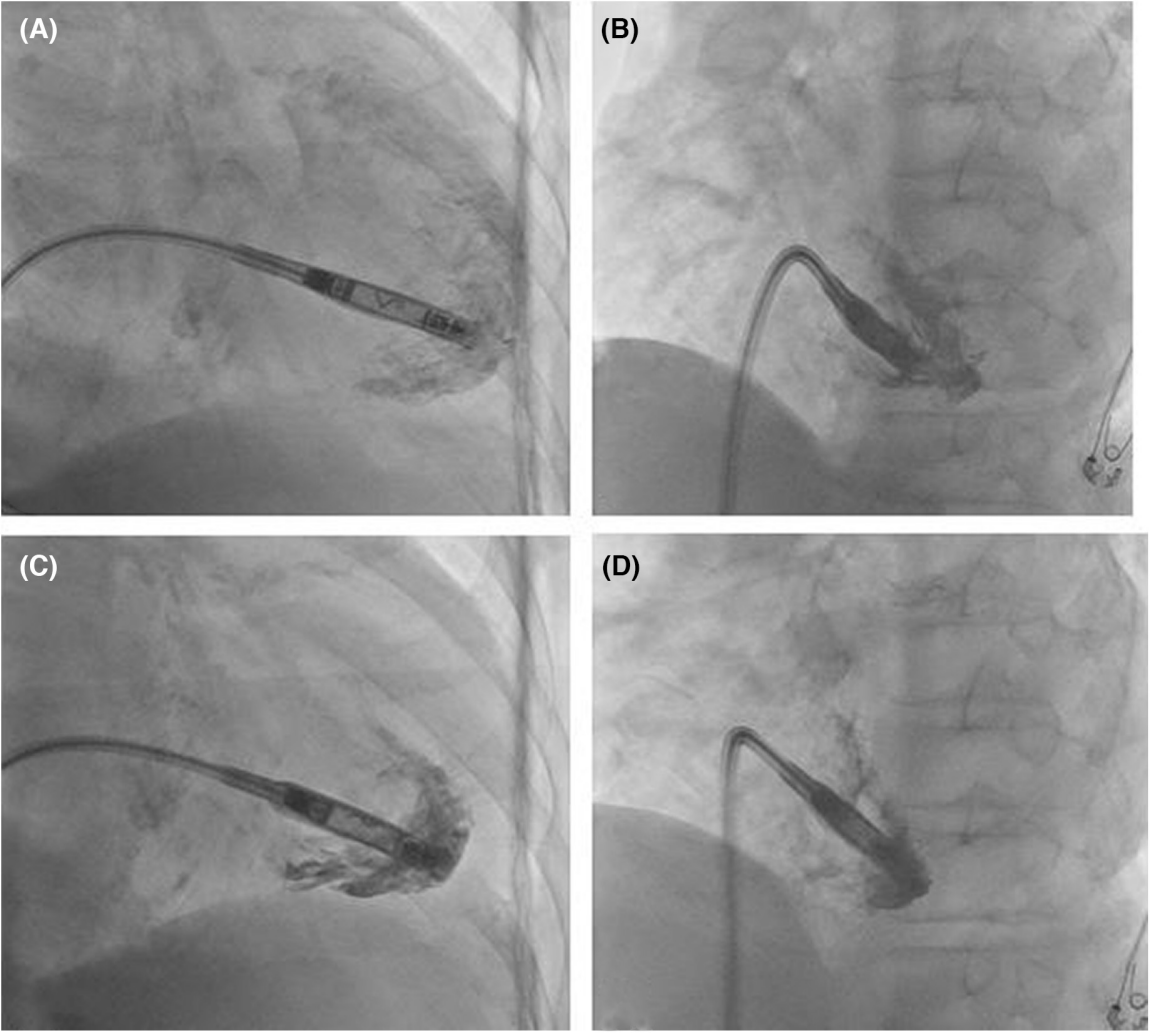
Fluoroscopic image of leadless pacemaker implantation in the (A) right anterior oblique (RAO) and (B) left anterior oblique (LAO) views at the first attempt. Before fixation parameters were the injury current was 3 mV, the threshold was 2.0 V at 0.4 ms, the R wave 3.0 mV, and the impedance 530 Ω. After fixation parameters were the injury current was 2 mV, the threshold was 2.5 V at 1.5 ms, the R wave 2.5 mV, and the impedance 880 Ω. Fluoroscopic image of leadless pacemaker implantation in the (C) RAO and (D) LAO views at the second attempt. Before fixation parameters were the injury current was 2 mV, the threshold was 0.75 V at 0.4 ms, the R wave 6.0 mV, and the impedance 520 Ω. After fixation parameters were the injury current was 2 mV, the threshold was 1.0 V at 0.4 ms, the R wave 6.0 mV, and the impedance 1010 Ω.

**FIGURE 2:**
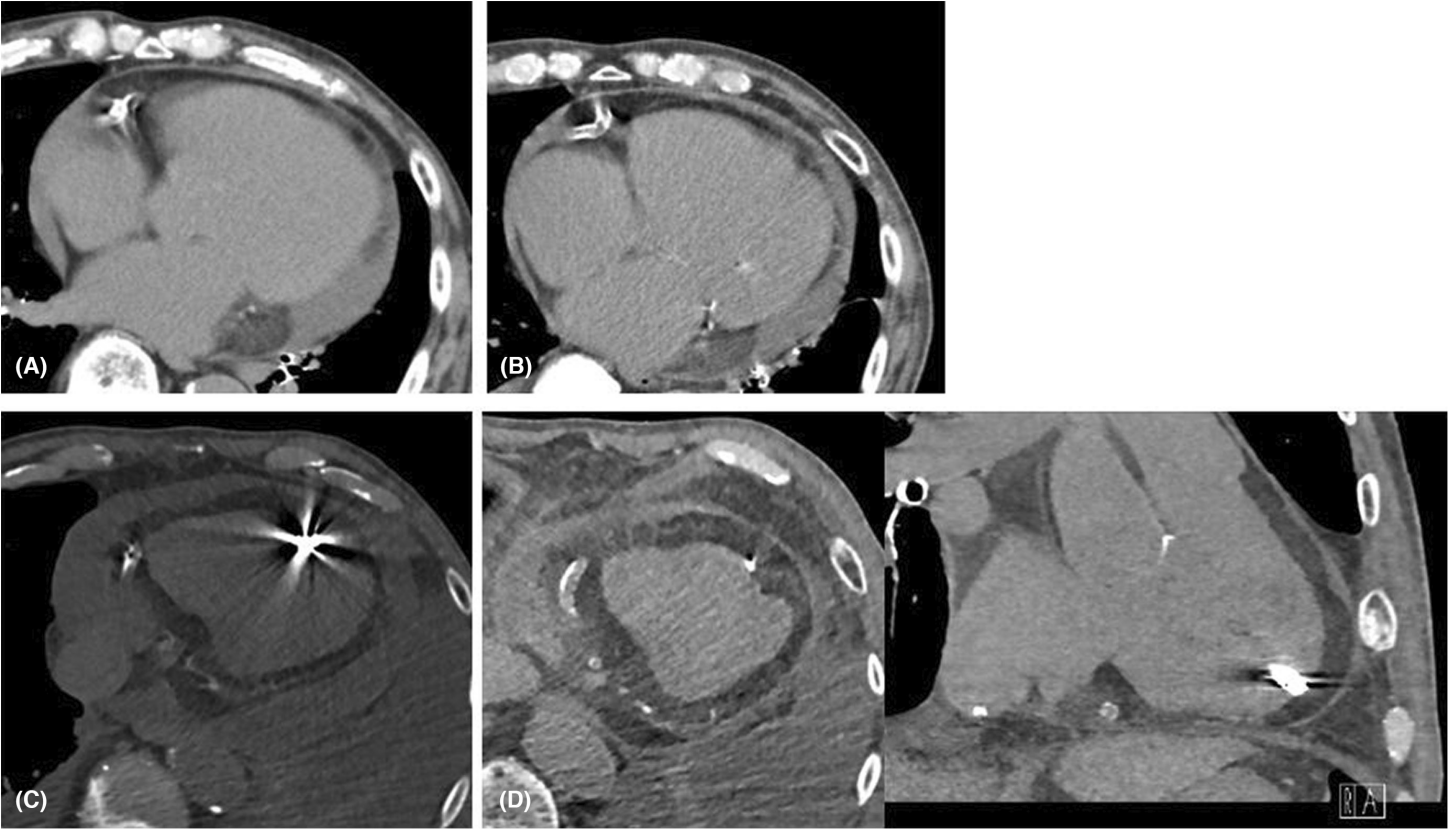
(A) Computed tomography (CT) image before leadless pacemaker implantation (LPI). CT shows pericardial effusion of unknown cause mainly in the posterior left ventricle. (B) CT image of the LP 1 month after LPI. (C) The LP at re‐admission of late cardiac tamponade. (D) The LP tip after pericardial drain removal (left panel; axial view; right panel, coronal plane).

However, in the Micra™ (Medtronic), an increased number of deployments and validation of a risk score for predicting pericardial effusion were reported.^1^ In this case, the patient was on hemodialysis and was at medium risk. Medium‐risk cases are associated with a risk of pericardiac effusion because of the increased number of deployments, and the Aveir‐VR™ had more successful single‐attempt deployments than the Micra™^2^; thus, we should have aimed for successful single‐attempt deployment. In addition, it is difficult to implant the Aveir‐VR™ on the septum because the Aveir‐VR™ is 38 mm long, which is too long to be implanted in the right ventricle without interfering with the tricuspid valve, especially in the Japanese population. The Aveir‐VR™ should be implanted closer to the apex when it is difficult to implant on the septum. Delayed cardiac lead perforation with the transvenous lead was reported to be more common with the active fixation lead such as the screw type than with the passive fixation lead such as the tined lead.^3^ Moreover, the incidence of a late cardiac tamponade should be monitored with the Aveir‐VR™ rather than with the Micra™. To prevent cardiac tamponade including late phase, identification of a safe anatomic location (closer to the septal site) using contrast imaging, confirmation of good electrical pre‐mapping, and slow rotation of the device within 1.25–1.5 clockwise turns are required when using the Aveir‐VR™ system to minimize the number of deployments and to aim for successful single‐attempt deployment.

## CONFLICT OF INTEREST STATEMENT

Authors declare no conflict of interests for this article.

## ETHICS STATEMENT

Approval was obtained from the local ethics committee.

## PATIENT CONSENT STATEMENT

Written informed consent was obtained from the patient for publication of this case report.
